# Trend and determinants of mortality in incident hemodialysis patients of the Lazio region

**DOI:** 10.1186/s12882-023-03170-w

**Published:** 2023-04-26

**Authors:** Maurizio Bossola, Anteo Di Napoli, Laura Angelici, Anna Maria Bargagli, Silvia Cascini, Ursula Kirchmayer, Nera Agabiti, Marina Davoli, Claudia Marino

**Affiliations:** 1 Servizio Emodialisi, Università Cattolica del Sacro Cuore facoltà di Medicina e Chirurgia, Rome, Italy; 2 Servizio Emodialisi, Fondazione Policlinico Universitario Agostino Gemelli IRCCS, Rome, Italy; 3 National Institute for Health, Migration and Poverty, 00153 Rome, Italy; 4 Department of Epidemiology, Regional Health Service – Lazio, Via Cristoforo Colombo, 112, 00147 Rome, Italy

**Keywords:** End-stage renal disease, Incident hemodialysis patients, Mortality trend.

## Abstract

**Background:**

. In the last decades some studies observed a moderate progressive decrease in short-term mortality in incident hemodialysis patients. The aim of the study is to analyse the mortality trends in patients starting hemodialysis using the Lazio Regional Dialysis and Transplant Registry.

**Methods:**

. Patients who started chronic hemodialysis between 2008 and 2016 were included. Annual 1-year and 3-year Crude Mortality Rate*100 Person Years (CMR*100PY) overall, by gender and age classes were calculated. Cumulative survival estimates at 1 year and 3 years since the date of starting hemodialysis were presented as Kaplan-Meier curves for the three periods and compared using the log-rank test. The association between periods of incidence in hemodialysis and 1-year and 3-year mortality were investigated by means of unadjusted and adjusted Cox regression models. Potential determinants of both mortality outcomes were also investigated.

**Results:**

. Among 6,997 hemodialysis patients (64.5% males, 66.1% over 65 years old) 923 died within 1 year and 2,253 within 3 years form incidence; CMR*100PY were 14.1 (95%CI: 13.2–15.0) and 13.7 (95%CI: 13.2–14.3), respectively; both remained unchanged over the years. Even after stratification by gender and age classes no significant changes emerged. Kaplan-Meier mortality curves did not show any statistically significant differences in survival at 1 year and 3 years from hemodialysis incidence across periods. No statistically significant associations were found between periods and 1-year and 3-year mortality. Factors associated with a greater increase in mortality are: being over 65 years, born in Italy, not being self-sufficient, having systemic versus undetermined nephropathy, having heart disease, peripheral vascular disease, cancer, liver disease, dementia and psychiatric illness, and receiving dialysis by catheter rather than fistula.

**Conclusions:**

. The study shows that the mortality rate in patients with end-stage renal disease starting hemodialysis in the Lazio region was stable over 9 years.

**Supplementary Information:**

The online version contains supplementary material available at 10.1186/s12882-023-03170-w.

## Background

Approximately, 65,000 end-stage renal disease (ESRD) patients start chronic hemodialysis (HD) every year, in Europe [1]. These patients are at a high risk of early and midterm death, the 1-year and 3-year mortality ranging between 10 and 17% and 32–45%, respectively [1–9].

Early and mid-term death after initiating dialysis is therefore a relevant problem that needs to be accounted for before renal replacement therapy becomes necessary and discussed with patients and families.

In the last 20 years, there have been significant advances in technology and the quality of chronic dialysis procedures and improvements in managing anemia, arterial hypertension, vascular access, and uremia/replacement therapy-related complications, such as osteodystrophy and cardiovascular diseases [10, 11].

At the same time, the proportion of incident patients aged over 65 years in Europe increased from 22% to 1980 to 55% in 2005, according to the European Renal Association Dialysis and Transplantation (ERA-EDTA) [12, 13], with an increase in the patients with age-related vascular nephropathies and diabetic nephropathy [14].

Interestingly, in the last few decades, a moderate progressive increase of short-term survival in incident HD patients has been observed over time in some studies [1–7] but not in others [4, 8, 9]. However, when present, the increase of survival was independent of the age of the patients’ population [1–7]. In a study conducted, using the Lazio Regional Dialysis and Transplant Registry (RRDTL) data, on a 1995–2006 cohort (about 9,000 subjects), the overall mortality rate remained essentially unchanged over the 12-year period, despite a 5-year increase in the median age, without statistically significant differences over time in annual mortality rates by cause of death [4].

In addition, a reduction over time of in excess mortality for atheromatous cardiovascular disease and infections has been reported, possibly due to the better management of dialysis patients with myocardial infarction or stroke in the last decades and better prevention of infections [1].

In this study, using the RRDTL, trends over time in 1-year and 3-year mortality were examined in ESRD patients starting chronic HD in Lazio, a region in central Italy that includes Rome, and has 5,5 million residents.

## Methods

### Aims

The present study aimed to analyse mortality within 1 year and 3 years from incidence in chronic HD among patients residing in Lazio region.

In detail objectives were:

1. To calculate the annual 1-year and 3-year mortality rate over a period of 9 years overall and by gender and age classes;
2. To analyse the association between period of incidence in HD and 1-year and 3-year mortality;
3. To identify factors associated with 1-year and 3-year mortality.

### Source of the data

The source of the data is the RRDTL a population-based registry established in 1994. It collects detailed information on socio-demographic status, clinical characteristics, dialysis treatments, and drug therapy for all patients undergoing dialysis in all public and private accredited dialysis centers of the Lazio Region in Italy. All dialysis units have to register the information on their patients and to update information every 6-months. The dialysis units are required to communicate the date and the reason of dialysis treatment’s termination (kidney transplant, renal recovery, transfer to another dialysis centers, death) [15, 16]. Details on the RRDTL are reported elsewhere [4, 17–20]. The Lazio regional mortality registry was used to confirm the date of death registered in the RRDTL [21].

### Study population

This cohort study includes ESRD patients who started HD between January 2008 and December 2016 and were recorded in the RRDTL at incident date.

### Exclusion criteria

Patients less than 18 years old and with a follow-up period shorter than 91 days were excluded to avoid possible acute dialysis cases, which may have been incorrectly recorded as chronic at the RRDTL.

### Outcome

The outcomes are death within 1 year of dialysis and death within 3 years of dialysis.

### Follow-up periods

Each patient with at least 91 days of follow-up after the date of first dialysis treatment (index date) was followed from index date up to the first of the following: death, change in prognosis (transplantation, renal function recovery), loss to follow-up and the end of the study. The end of the study was 1 year and 3 years from the index date for 1 and 3-year outcomes respectively. Change in prognosis or loss to follow-up were reasons for censuring.

### Exposure (aim2)

Periods of incidence in HD: 2008–2010, 2011–2013, 2014–2016.

### Co-variates or potential risk factors

Variables recorded in the RRDTL were evaluate at the index date and were considered as co-variates for the aim 2 and as potential risk factors for the aim 3.

### Socio-demographic and clinical variables

The following socio-demographic variables were considered: gender, age classes (< 64, ≥ 65 years), place of birth (Italy, other countries), residence (Rome Municipality, Rome Province, Other Lazio’s Municipalities) and educational level (Up to Middle School, High School or more). Furthermore, were considered both clinical variables such as body mass index (BMI < 18.0, 18.0 ≤ BMI < 25.0, 25.0 ≤ BMI < 30.0, BMI ≥ 30.0), self-sufficiency (Total autonomy, Autonomy in some activities, Not self -sufficient), cause of ESRD (renal vascular disease, diabetic nephropathy, glomerulonephritis, cystic renal disease and familial nephropathy, interstitial and toxic nephritis/pyelonephritis, systemic disease, renal malformation, other nephropathies, unknown) and the following comorbidities: severe hypertension, heart disease, diabetes, peripheral vascular diseases, cerebrovascular disease, chronic obstructive pulmonary disease (COPD), cancer, thyroid disease, lipid metabolism’s alteration, motor deficit, liver disease, extrauremic anemia, dementia and psychiatric disease, malnutrition, obesity: (yes vs. no).

### Treatment variables

Use of drugs for diabetes, hypertension, other cardiovascular disease and anemia, and type of vascular access (catheter-CVC, fistula-FAV) evaluated at the index date.

### Care-related variables

Type of dialysis centre (public, private), pre-dialysis counselling (patients followed by nephrodialytic clinic in the 6 months prior to the start of dialysis; yes vs. no).

### Laboratory findings

The laboratory findings collected included: haemoglobin Hb (g/dL), calcium (mg/dL), serum creatinine (mg/dL), serum phosphate level (mg/dL), serum albumin (mg/dL) evaluated at the index date.

### Statistical analysis

Socio-demographic, clinical, treatment, care-related variables and laboratory findings were presented as percentage or mean [IQR], according to periods of incidence in dialysis and to vital status within 1 year and 3 years from index date. The relationship between categorical variables and period or vital status were tested by Chi-square test. The association between normally distributed continuous variables and period or vital status were tested by ANOVA or T-test respectively; the association with the other continuous variables were tested by non-parametric tests, namely Kruskal-Wallis or Wilcoxon rank sum tests.

Crude Mortality Rates per 100 Person Years (CMR*100PY) were calculate at 1 year and 3 years by calendar year of incidence (from 2008 to 2016), as the ratio of the number of deaths over the person time at risk. Plots of mortality rates across years were presented stratified by gender and age classes. For both outcomes JoinPoint models were performed to evaluate time trends in mortality rates overall and stratified by gender and age classes [22].

Cumulative survival estimates at 1 year and 3 years since index date were presented as Kaplan-Meier curves for the three periods and compared using the log-rank test. Kaplan-Meier curves were also presented according to age classes.

Furthermore, Cox univariate proportional hazards regression analyses were performed to explore the association between potential confounders and mortality within 1 year and 3 years since index date, and a multivariable Cox model was used to explore the association between periods of incidence and mortality in HD and the two outcomes adjusted for potential confounders.

Finally, Cox multivariable proportional hazards regression analyses were performed to identify factors associated with 1-year and 3-year mortality.

Variables significantly associated with the outcomes in the univariate analysis were introduced in the multivariate Cox models by a stepwise procedure. Comparative risk estimates were expressed as hazard ratios (HR) and 95% Confidence Interval (CI). Statistical significance was set at a 2-tailed p-value of 0.05.

A further analysis of the mortality outcome occurring after the first year from the index date within the third year was performed to evaluate the determinants of mortality in this specific period.

All data were analysed using SAS version 9.4 (SAS Institute, Cary, NC, USA).

## Results

In the cohort of 6,997 incident chronic HD patients during the period 2008–2016 the majority was male (64.5%), over 65 years old (66.1%), born in Italy (91.7%) and residing in the Municipality of Rome (50.2%).

### Characteristics of the study population by periods.

The baseline characteristics of the population at the index date according to periods of incidence in HD are shown in Table 1.

**Table 1:** Demographic and clinical characteristics of HD incident patients, by period of incidence in HD

	2008–2010			2011–2013			2014–2016			Total			p-value*
	N	%		N	%		N	%		N	%		
**Total**	2,317	100.0		2,295	100.0		2,385	100.0		6,997	100.0		
**Gender**													0.241
**Male**	1,469	63.4		1,478	64.4		1,568	65.7		4,515	64.5		
**Female**	848	36.6		817	35.6		817	34.3		2,482	35.5		
**Age (years)**													0.452
**< 65**	782	33.8		759	33.1		830	34.8		2,371	33.9		
**≥ 65**	1,535	66.3		1,536	66.9		1,555	65.2		4,626	66.1		
**Site of birth**													< 0.001
**Italy**	2,155	93.0		2,108	91.9		2,151	90.2		6,414	91.7		
**Other countries**	162	7.0		187	8.2		234	9.8		583	8.3		
**Residence**													0.825
**Rome Municipality**	1,149	49.6		1,149	50.1		1,216	51.0		3,514	50.2		
**Rome Province**	557	24.0		560	24.4		554	23.2		1,671	23.9		
**Other Lazio’s municipalities**	611	26.4		586	25.5		615	25.8		1,812	25.9		
**Education level**													< 0.001
**Up to Middle School**	1,622	70.0		1,520	66.2		1,504	63.1		4,646	66.4		
**High School or more**	695	30.0		775	33.8		881	36.9		2,351	33.6		
**Body Mass Index (Kg/m** ^2^ **)**													0.083
**Underweight (BMI < 18.0)**	106	4.6		117	5.1		128	5.4		351	5.0		
**Normal weight (18.0 ≤ BMI < 25.0)**	1,221	52.7		1,175	51.2		1,164	48.8		3,560	50.9		
**Overweight (25.0 ≤ BMI < 30.0)**	691	29.8		661	28.8		742	31.1		2,094	29.9		
**Obese (BMI ≥ 30.0)**	299	12.9		342	14.9		351	14.7		992	14.2		
**Self-sufficiency**													< 0.001
**Total autonomy**	839	36.2		1,028	44.8		1,348	56.5		3,215	45.9		
**Autonomy in some activities**	864	37.3		755	32.9		552	23.1		2,171	31.0		
**Not self-sufficient**	614	26.5		512	22.3		485	20.3		1,611	23.0		
**Nephropathy**													< 0.001
**Renal vascular disease**	586	25.3		567	24.7		508	21.3		1,661	23.7		
**Diabetic nephropathy**	575	24.8		527	23.0		525	22.0		1,627	23.3		
**Glomerulonephritis**	204	8.8		182	7.9		190	8.0		576	8.2		
**Cystic renal disease and familial nephropathy**	149	6.4		162	7.1		164	6.9		475	6.8		
**Interstitial and toxic nephritis/pyelonephriti**	133	5.7		135	5.9		123	5.2		391	5.6		
**Systemic disease**	82	3.5		58	2.5		70	2.9		210	3.0		
**Renal malformation**	12	0.5		14	0.6		6	0.3		32	0.5		
**Other nephropathies**	83	3.6		99	4.3		132	5.5		314	4.5		
**Unknown**	493	21.3		551	24.0		667	28.0		1,711	24.5		
**Comorbidity**													
**Severe hypertension**	1,478	63.8		1,524	66.4		1,665	69.8		4,667	66.7		< 0.001
**Heart disease**	854	36.9		862	37.6		859	36.0		2,575	36.8		0.548
**Diabetes**	605	26.1		661	28.8		690	28.9		1,956	28.0		0.054
**Peripheral vascular diseases**	359	15.5		351	15.3		327	13.7		1,037	14.8		0.168
**Cerebrovascular disease**	301	13.0		350	15.3		321	13.5		972	13.9		0.064
**COPD**	316	13.6		319	13.9		336	14.1		971	13.9		0.905
**Cancer**	227	9.8		236	10.3		296	12.4		759	10.8		0.009
**Thyroid disease**	138	6.0		153	6.7		230	9.6		521	7.4		< 0.001
**Obesity**	138	6.0		165	7.2		198	8.3		501	7.2		0.008
**Lipid metabolism’s alteration**	151	6.5		153	6.7		166	7.0		470	6.7		0.826
**Liver disease**	84	3.6		82	3.6		86	3.6		252	3.6		0.995
**Motor deficit**	66	2.9		79	3.4		74	3.1		219	3.1		0.509
**Dementia and Psychiatric disease**	63	2.7		63	2.8		89	3.7		215	3.1		0.072
**Extrauremic anemia**	68	2.9		56	2.4		62	2.6		186	2.7		0.566
**Malnutrition**	35	1.5		53	2.3		50	2.1		138	2.0		0.129
**Drugs use**													
**Diabetes drugs**	596	25.7		643	28.0		633	26.5		1,872	26.8		0.204
**Hypertensive drugs**	1,932	83.4		1,948	84.9		2,039	85.5		5,919	84.6		0.121
**Other cardiovascular drugs**	1,502	64.8		1,508	65.7		1,550	65.0		4,560	65.2		0.799
**Anti-anaemic drugs**	2,072	89.4		2,041	88.9		2,115	88.7		6,228	89.0		0.708
**Vascular access**													< 0.001
**CVC**	988	42.6		1,117	48.7		1,258	52.8		3,363	48.1		
**FAV**	1,329	57.4		1,178	51.3		1,127	47.3		3,634	51.9		
**Type of dialysis centre**													< 0.001
**Public**	1,238	53.4		1,100	47.9		1,228	51.5		3,566	51.0		
**Private**	1,079	46.6		1,195	52.1		1,157	48.5		3,431	49.0		
**Pre-dialysis counselling**													< 0.001
**Yes**	1,808	78.0		1,816	79.1		1,711	71.7		5,335	76.2		
**No**	509	22.0		479	20.9		674	28.3		1,662	23.8		
**Laboratory findings**	**mean [IQR]**			**mean [IQR]**			**mean [IQR]**			**mean [IQR]**			**p-value^**
**HB (g/dL)**	10.4 [9.4–11.2]			10.3 [9.4–11.2]			10.2 [9.1–11.0]			10.2 [9.1–11.0]			< 0.001
**Calcium (mg/dL)**	8.7 [8.2–9.2]			8.7 [8.2–9.1]			8.7 [8.2–9.1]			8.7 [8.2–9.1]			0.025
**Serum creatinine (mg/dL)**	7.0 [5.4–8.2]			6.7 [5.1–7.9]			6.8 [5.1-8.0]			6.8 [5.1-8.0]			< 0.001
**Serum phosphate level (mg/dL)**	5.1 [4.2–5.9]			5.0 [4-5.8]			4.9 [4.0-5.7]			4.9 [4.0-5.7]			< 0.001
**Serum albumin (mg/dL)**	3.7 [3.4-4.0]			3.6 [3.3-4]			3.5 [3.2–3.9]			3.5 [3.2–3.9]			< 0.001

*χ2 test or Fisher’s exact for categorical variables

^Anova or Kruskal-Wallis test for continuous variables

Statistically significant differences between periods were found for site of birth, educational level and self-sufficiency, respectively with a higher percentage of patients born abroad of Italy (9.8% vs. 7.0%), with a higher level of education (36.9% vs. 30.0%) and totally autonomous patients (56.5% vs. 36.2%), in the third period respect to the first one. The prevalence of comorbidities such as severe hypertension, cancer, thyroid disease and obesity increased in a statistically significant way across periods (Table 1). A lower percentage of FAV (47.3% vs. 57.4%) and of patients receiving pre-dialysis counselling (71.7% vs. 78.0%) was found in the third period compared to the first one. A higher percentage of patients undergoing dialysis in private clinic (48.5% vs. 46.6%) was found in the third period compared to the first one. Finally, statistically significant differences between periods were found for all laboratory findings.

### Characteristics of the study population by vital status

Table 2 shows characteristics of the study population by vital status at 1 year and 3 years and their corresponding unadjusted cause-specific HRs along with 95%CI.

**Table 2:** Demographic and clinical characteristics of HD patients by vital status for 1 and 3-year mortality and unadjusted cause-specific HR.

				1 year Mortality						3 year Mortality						
		Died		Alive		Unadjusted cause-specific HR	95% CI			Died		Alive		Unadjusted cause-specific HR	95% CI	
		N	%	N	%		lower	upper		N	%	N	%		lower	upper
**Total**		923	100.0	6,074	100.0					2,253	100.0	4,744	100.0			
**Period**																
** 2008–2010**		294	31.9	2,023	33.3	ref				739	32.8	1,578	33.3	ref		
** 2011–2013**		305	33.0	1,990	32.8	1.05	0.90	1.23		750	33.3	1,545	32.6	1.01	0.92	1.12
** 2014–2016**		324	35.1	2,061	33.9	1.07	0.92	1.26		764	33.9	1,621	34.2	0.99	0.89	1.09
**Gender**																
** Male**		575	62.3	3,940	64.9					1,466	65.1	3,049	64.3	ref		
** Female**		348	37.7	2,134	35.1	1.11	0.97	1.27		787	34.9	1,695	35.7	0.98	0.90	1.07
**Age (years)**																
** < 65**		149	16.1	2,222	36.6	ref				366	16.3	2,005	42.3	ref		
** ≥ 65**		774	83.9	3,852	63.4	2.78	2.33	3.31		1,887	83.8	2,739	57.7	2.95	2.64	3.30
**Site of birth**																
** Italy**		900	97.5	5,514	90.8	3.75	2.48	5.66		2,179	96.7	4,235	89.3	3.06	2.42	3.85
** Other countries**		23	2.5	560	9.2	ref				74	3.3	509	10.7	ref		
**Residence**																
** Rome Municipality**		414	44.9	3,100	51.0	ref				1,043	46.3	2,471	52.1	ref		
** Rome Province**		246	26.7	1,425	23.5	1.27	1.09	1.49		582	25.8	1,089	23.0	1.22	1.10	1.35
** Other Lazio’s municipalities**		263	28.5	1,549	25.5	1.25	1.07	1.45		628	27.9	1,184	25.0	1.20	1.08	1.32
**Educational level**																
** Up to Middle School**		681	73.8	3,965	65.3	1.45	1.25	1.68		1,636	72.6	3,010	63.5	1.39	1.26	1.52
** High School or more**		242	26.2	2,109	34.7	ref				617	27.4	1,734	36.6	ref		
**Body Mass Index (Kg/m** ^2^ **)**																
** Underweight (BMI < 18.0)**		83	9.0	268	4.4	1.76	1.39	2.21		152	6.8	199	4.2	1.44	1.22	1.70
** Normal weight (18.0 ≤ BMI < 25.0)**		505	54.7	3,055	50.3	ref				1,186	52.6	2,374	50.0	ref		
** Overweight (25.0 ≤ BMI < 30.0)**		249	27.0	1,845	30.4	0.82	0.71	0.96		646	28.7	1,448	30.5	0.89	0.81	0.98
** Obese (BMI ≥ 30.0)**		86	9.3	906	14.9	0.59	0.47	0.74		269	11.9	723	15.2	0.74	0.65	0.85
**Self-sufficiency**																
** Total autonomy**		177	19.2	3,038	50.0	ref				572	25.4	2,643	55.7	ref		
** Autonomy in some activities**		294	31.9	1,877	30.9	2.56	2.12	3.08		767	34.0	1,404	29.6	2.18	1.96	2.43
** Not self-sufficient**		452	49.0	1,159	19.1	5.82	4.89	6.92		914	40.6	697	14.7	4.36	3.93	4.84
**Nephropathy**																
** Renal vascular disease**		228	24.7	1,433	23.6	0.93	0.78	1.12		589	26.1	1,072	22.6	0.97	0.87	1.09
** Diabetic nephropathy**		240	26.0	1,387	22.8	1.00	0.84	1.20		580	25.7	1,047	22.1	0.99	0.88	1.11
** Glomerulonephritis**		31	3.4	545	9.0	0.36	0.25	0.53		88	3.9	488	10.3	0.41	0.32	0.51
** Cystic renal disease and familial nephropathy**		22	2.4	453	7.5	0.30	0.20	0.47		66	2.9	409	8.6	0.35	0.27	0.45
** Interstitial and toxic nephritis/pyelonephriti**		54	5.9	337	5.6	0.96	0.71	1.29		119	5.3	272	5.7	0.85	0.70	1.04
** Systemic disease**		47	5.1	163	2.7	1.67	1.22	2.28		88	3.9	122	2.6	1.34	1.07	1.67
** Renal malformation**		4	0.4	28	0.5	0.84	0.31	2.25		6	0.3	26	0.6	0.50	0.22	1.11
** Other nephropathies**		48	5.2	266	4.4	1.07	0.78	1.46		110	4.9	204	4.3	1.01	0.83	1.24
** Unknown**		249	27.0	1,462	24.1	ref				607	26.9	1,104	23.3	ref		
**Comorbidity (HR: Yes vs. No)**																
** Severe hypertension**		521	56.5	4,146	68.3	0.62	0.54	0.71		1,356	60.2	3,311	69.8	0.69	0.63	0.75
** Heart disease**		479	51.9	2,096	34.5	1.95	1.71	2.21		1,126	50.0	1,449	30.5	1.93	1.77	2.09
** Diabetes**		300	32.5	1,656	27.3	1.25	1.09	1.44		718	31.9	1,238	26.1	1.24	1.13	1.35
** Peripheral vascular diseases**		221	23.9	816	13.4	1.92	1.65	2.23		460	20.4	577	12.2	1.65	1.49	1.83
** Cerebrovascular disease**		179	19.4	793	13.1	1.55	1.32	1.83		443	19.7	529	11.2	1.66	1.50	1.84
** COPD**		194	21.0	777	12.8	1.72	1.47	2.02		459	20.4	512	10.8	1.77	1.60	1.96
** Cancer**		164	17.8	595	9.8	1.87	1.58	2.22		349	15.5	410	8.6	1.72	1.53	1.92
** Thyroid disease**		74	8.0	447	7.4	1.09	0.86	1.38		169	7.5	352	7.4	1.01	0.87	1.18
** Obesity**		57	6.2	444	7.3	0.84	0.64	1.09		152	6.8	349	7.4	0.89	0.75	1.05
** Lipid metabolism’s alteration**		48	5.2	422	7.0	0.75	0.56	1.00		120	5.3	350	7.4	0.74	0.61	0.89
** Liver disease**		56	6.1	196	3.2	1.88	1.43	2.46		103	4.6	149	3.1	1.50	1.23	1.83
** Motor deficit**		56	6.1	163	2.7	2.18	1.67	2.86		113	5.0	106	2.2	1.94	1.61	2.35
** Dementia and Psychiatric disease**		53	5.7	162	2.7	2.05	1.55	2.70		113	5.0	102	2.2	2.02	1.67	2.44
** Extrauremic anemia**		45	4.9	141	2.3	2.04	1.51	2.76		73	3.2	113	2.4	1.36	1.08	1.72
** Malnutrition**		37	4.0	101	1.7	2.28	1.64	3.16		66	2.9	72	1.5	1.79	1.40	2.28
**Drugs use (HR: Yes vs. No)**																
** Diabetes drugs**		280	30.3	1,592	26.2	1.20	1.04	1.38		688	30.5	1,184	25.0	1.23	1.12	1.34
** Hypertensive drugs**		744	80.6	5,175	85.2	0.74	0.63	0.87		1,868	82.9	4,051	85.4	0.83	0.75	0.93
** Other cardiovascular drugs**		625	67.7	3,935	64.8	1.11	0.97	1.28		1,577	70.0	2,983	62.9	1.25	1.14	1.37
** Anti-anaemic drugs**		851	92.2	5,377	88.5	1.50	1.18	1.91		2,041	90.6	4,187	88.3	1.24	1.07	1.43
**Vascular access**																
** CVC**		665	72.1	2,698	44.4	3.05	2.64	3.52		1,390	61.7	1,973	41.6	2.09	1.92	2.27
** FAV**		258	28.0	3,376	55.6	ref				863	38.3	2,771	58.4	ref		
**Type of dialysis centre**																
** Public**		477	51.7	3,089	50.9	ref				1,177	52.2	2,389	50.4	ref		
** Private**		446	48.3	2,985	49.1	0.96	0.85	1.09		1,076	47.8	2,355	49.6	0.94	0.87	1.02
**Pre-dialysis counselling**																
** No**		231	25.0	1,431		ref				507	22.5	1,155	24.4	ref		
** Yes**		692	75.0	4,643	76.4	0.92	0.79	1.06		1,746	77.5	3,589	75.7	1.05	0.95	1.16
		**mean [IQR]**		**mean [IQR]**						**mean [IQR]**		**mean [IQR]**				
**Laboratory findings, mean [IQR]**																
** HB (g/dL)**		10.0 [9.0-10.8]		10.3 [9.4–11.2]		0.82	0.78	0.86		10.2 [9.2–11]		10.4 [9.4–11.2]		0.90	0.88	0.93
** Calcium (mg/dL)**		8.6 [8.0–9.0]		8.7 [8.2–9.2]		0.86	0.79	0.93		8.6 [8.1–9.1]		8.7 [8.2–9.2]		0.91	0.87	0.96
** Serum creatinine (mg/dL)**		6.1 [4.6–7.5]		6.9 [5.3–8.2]		0.84	0.82	0.87		6.2 [4.7–7.5]		7.1 [5.5–8.3]		0.85	0.84	0.87
** Serum phosphate level (mg/dL)**		4.7 [3.8–5.5]		5.0 [4.1–5.8]		0.86	0.82	0.90		4.8 [3.9–5.6]		5.1 [4.1–5.9]		0.87	0.84	0.89
** Serum albumin (mg/dL)**		3.4 [3.1–3.8]		3.6 [3.3-4.0]		0.47	0.42	0.53		3.5 [3.2–3.9]		3.7 [3.4-4]		0.60	0.56	0.65

^HR = hazard ratio

### 1-year mortality

923 out of 6,997 died within 1 year of follow-up; the percentage of censored subjects during the first year of dialysis was 3% of which 1% refers to patients who received renal transplantation; those percentages were similar during the three periods of study. One-year crude mortality rates per 100 Person Years in the three periods were 2008–2010: 13.5 (95%CI: 12.1–15.2); 2011–2013: 14.2 (95%CI: 12.7–15.9); 2014–2016: 14.5 (95%CI: 13.0-16.2).

One-year mortality was higher for subjects over 65 vs. under 65 years (HR: 2.78, 95%CI: 2.33–3.31), born in Italy vs. born in other countries (HR: 3.75, 95%CI: 2.48–5.66), resident in Rome Province and in Other Lazio’s municipalities vs. resident in Rome municipality (HR: 1.27, 95%CI: 1.09–1.49 and 1.25, 95%CI: 1.07–1.45, respectively), with a low level of education vs. high (HR: 1.45, 95%CI: 1.25–1.68), BMI < 18.0 kg/m^2^ vs. normal weight (HR: 1.76, 95%CI: 1.39–2.21), not self-sufficient vs. total autonomy (HR: 5.82, 95%CI: 4.89–6.92). The nephropathy with the highest 1-year mortality was the systemic one vs. unknown nephropathy (HR: 1.67 95%CI: 1.22–2.28), and the HRs for comorbidities significantly associated with 1-year mortality ranged from 0.62 for severe hypertension to 2.28 for malnutrition. Finally, 1-year mortality was higher for users of diabetes drugs (HR: 1.20, 95%CI: 1.04–1.38), for users of anti-anaemic drugs (HR: 1.50, 95%CI: 1.18–1.91) and for subjects with a CVC as vascular access vs. FAV (HR: 3.05, 95%CI: 2.64–3.52).

### 3-year mortality

2,253 out of 6,997 died within 3 years of follow-up; the percentage of censored subjects during the 3 years of dialysis was 9% of which 5% refer to patients who received renal transplantation; those percentages were similar during the three periods of study. Three-year crude mortality rates per 100 Person Years in the three periods were 2008–2010: 13.7 (95% CI: 12.8–14.7); 2011–2013: 13.9(95% CI: 12.9–14.9); 2014–2016: 13.5(95%CI: 12.6–14.5). Three-year mortality was higher for subjects older than 65 years vs. younger (HR: 2.95, 95%CI: 2.64–3.30), born in Italy vs. born in other countries (HR: 3.06, 95%CI:2.42–3.85), resident in Rome Province and in Other Lazio’s municipalities vs. resident in Rome municipality (HR: 1.22, 95%CI: 1.10–1.35 and 1.20, 95%CI: 1.08–1.32, respectively) with a low level of education vs. high (HR: 1.39, 95%CI: 1.26–1.52), BMI < 18.0 kg/m^2^ vs. normal weight (HR: 1.44, 95%CI: 1.22–1.70), and not self-sufficient vs. total autonomy (HR: 4.36, 95%CI: 3.93–4.84). The nephropathy with the highest 3-year mortality was the systemic one vs. unknown nephropathy (HR: 1.34, 95%CI: 1.07–1.67), and the HRs for the comorbidities significantly associated with 3-year mortality ranged from 0.69 for severe hypertension to 2.02 for dementia and psychiatric disease. Finally, 3-year mortality was higher for users of antidiabetic drugs (HR: 1.23, 95%CI: 1.12–1.34), for users of other cardiovascular drugs (HR: 1.25, 95%CI: 1.14–1.37) and for users of anti-anaemic drugs (HR: 1.24, 95%CI: 1.07–1.43), and for subjects with a CVC as vascular access vs. FAV (HR: 2.09, 95%CI: 1.92–2.27).

### Mortality annual trend

**Figure 1:**
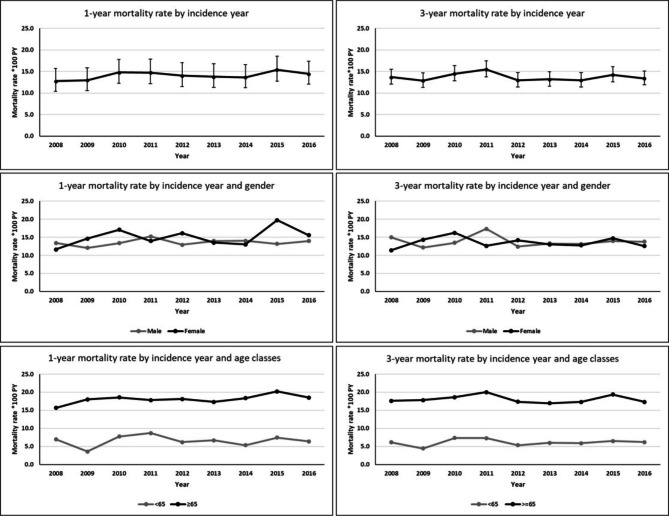
Crude 1-year and 3-year mortality rates by calendar years of incidence in HD

Figure 1 shows the trend of the crude annual 1-year and 3-year mortality rate by year of incidence, gender and age classes. The crude mortality rate remained largely unchanged over the years with small annual fluctuations in both 1-year and 3-year mortality. Even after stratification by gender and age classes no significant changes emerged over the years. The JoinPoint analysis confirmed the absence of any trends in annual rates for the two outcomes, both with the assumption of zero joinpoint and with the assumption of one joinpoint. (Supplementary Figure S1)

### Association between periods of incidence in HD and 1- year and 3-year mortality

Kaplan-Meir curves show the absence of a statistically significant differences in survival at 1 year (log-rank test p = 0.666) and 3 years (log-rank test p = 0.882) since HD incidence across the three considered periods. The same results were confirmed when stratifying by age groups (survival at 1 year < 65: log-rank test p = 0.705, ≥ 65: log-rank test p = 0. 563; survival at 3 years < 65: log-rank test p = 0.955, ≥ 65: log-rank test p = 0.990). The 1-year survival probability was: 0.94 (95%CI: 0.93–0.95) in age class < 65 years and 0.83 (95%CI: 0.82–0.84) in age class ≥ 65 years, this difference increased considering 3-year survival: 0.83 (95%CI: 0.81–0.85) and 0.58 (95%CI: 0.57–0.59) respectively. Kaplan-Meir curves are shown in Supplementary Figure S2.

Variables found to be significantly associated with mortality at 1 year and 3 years and included in the multivariate analysis were: age at incidence, place of birth, residence, BMI, self-sufficiency, cause of ESRD, severe hypertension, heart disease, cancer, peripheral vascular diseases, liver disease, dementia and psychiatric disease, type of vascular access, HB, serum creatinine, serum albumin. For mortality at 3 years COPD and lipid metabolism’s alteration were also found.

In the adjusted Cox regression model for 1-year mortality HRs of 1.10 95%CI:0.93–1.29 and 1.06 95%CI:0.90–1.25 were found, respectively in periods 2011–2013 and 2014–2016 compared to the reference period 2008–2010.

Similar results were obtained for 3-year mortality with HRs of 1.05 95%CI:0.94–1.17 and 1.03 95%CI: 0.93–1.14, respectively in periods 2011–2013 and 2014–2016 compared to the reference period 2008–2010.

### Factors associated with 1- year and 3-year mortality

**Figure 2:**
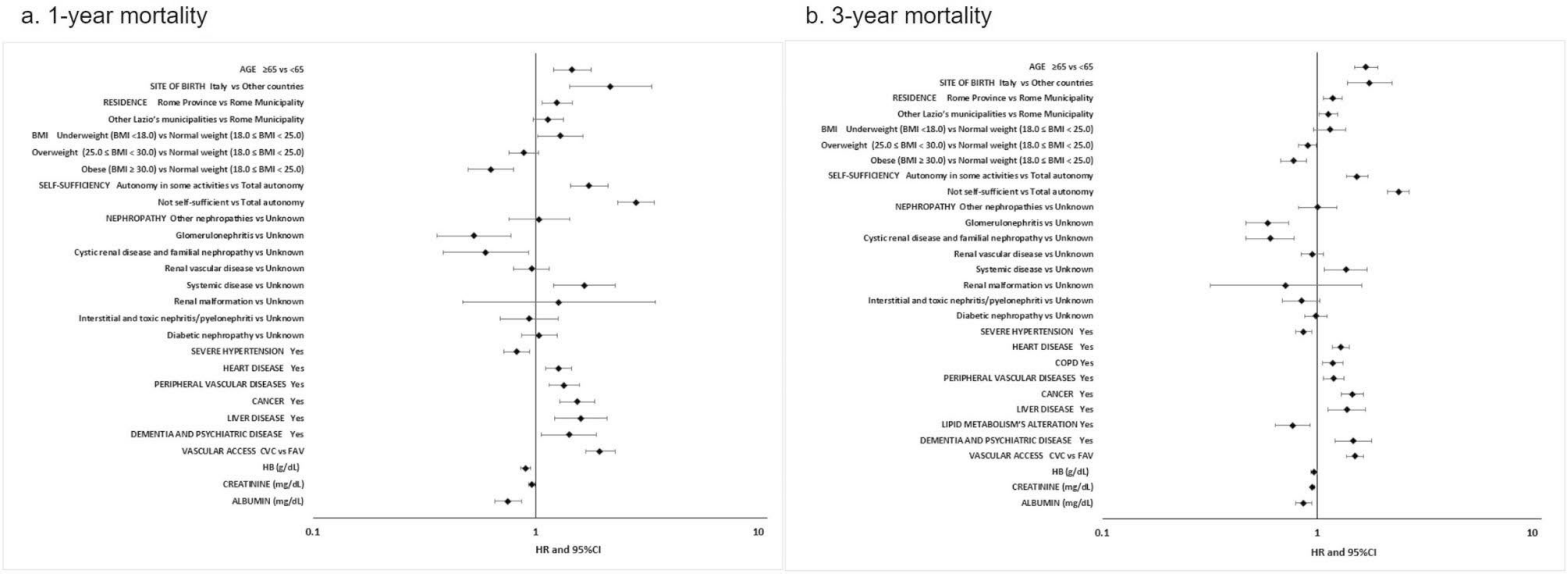
Factors associated within 1 and 3 years from incidence in HD. Multivariable Cox regression analysis

After stepwise regression factors associated with 1-year and 3-year mortality were: age at incidence, place of birth, residence, BMI, self-sufficiency, cause of ESRD, severe hypertension, heart disease, peripheral vascular diseases, cancer, liver disease, dementia and psychiatric disease, type of vascular access, haemoglobin, serum creatinine, serum albumin. COPD and lipid metabolism’s alteration were included as covariates only in the 3-year mortality Cox model. HR and 95% CIs are reported in Fig. 2.

The results for the mortality outcome occurring after the first year from the index date within the third year were presented in Supplementary Table S1.

## Discussion

The present study showed that the 1-year and 3-year mortality in incident HD patients was stable over a period of 9 years, since 2008 to 2016 in Lazio, a central region of Italy including the city of Rome. This observation is confirmed both by Kaplan-Meier survival curves, also after stratification by age and gender, and by raw and adjusted Cox regression models. In the light of an increasing complexity of patients starting HD over time, this is a positive signal, which confirms the consistent quality of HD assistance in Lazio.

Nevertheless, about 14% and 35% of incident HD patients died within the first and third year since the start of HD, in the three calendar periods, respectively, and the hypothesis of increasing survival as a consequence of advances in clinical care and specifically of improvements of dialysis techniques and materials, new medications, increase of life expectancy, better management of comorbidities and malnutrition, better prevention of infections and an uptake of clinical practice guidelines, occurring over time, was not met.

Using a different analytic approach, two Registry studies have demonstrated, recently, that mortality rates decreased significantly over calendar periods in patients initiating HD. Foster et al. [7] have demonstrated that among almost 2 million individuals initiating ESRD care in the United States from 1995 to 2013, the excess risk of ESRD-related compared relative to the general population decreased significantly with advancing calendar time. Similarly, data from the ERA-EDTA Registry show that, between 2007 and 2015, in patients initiating HD, the excess mortality rate decreased from 178 extra deaths per 1000 person-year to 154 extra deaths per 1,000 person-years [1]. In the UK Registry essentially no change was observed in the death rate from 2013 to 2014 on a background of a declining trend in the death rate overall and over the past decade [9].

The lack of improvement in 1-year and 3-year mortality in the period 2008–2016 in the Lazio incident HD patients is difficult to explain, taking into consideration that the survival had an about 1-year improvement in the general population in the same period, either in Lazio and in Italy [23] and considering that the dialysis centres of Lazio included in the RRDTL follow the international and national guidelines for the treatment of ESRD patients and offer every advance in patient care. Moreover, unvaried mortality trends were observed in all ages (< 65 years, ≥ 65 years), which is in disagreements with findings from other studies. Foster et al. [7] reported that younger incident HD patients showed significant larger relative improvements in excess mortality than older people and Boenink et al. [1] demonstrated that the strongest decrease in excess mortality was observed in patients ≥ 65 years, with smaller reductions among the youngest patients. Accordingly, in the UK registry, the death rate per 1,000 patient years in the first year of starting HD from 2003 to 2012 showed a declining trend with a larger rate of decline in patients aging ≥ 65 years [9]. We underline that the purpose of our study is to assess the trend and determinants of mortality in HD, not to make comparisons between regions. However, comparisons with mortality rates observed from other registries should be made with caution, as there may be a different case mix of people on HD. In particular, the lower renal transplant and peritoneal dialysis supply rates may result in younger, healthier individuals among HD patients in Lazio. Furthermore, the rates of transplantation and peritoneal dialysis incidence in Lazio did not change substantially during the period of the study, with annul standardised kidney transplant rates per 100,000 ranging from 2.4 to 3.6 in the period 2008–2017 [24] and the proportion of patients treated with peritoneal dialysis among dialysis patients range from 8 to 13% [16]. Therefore, it is unlikely that the potential selection bias could have affected the analyses and the conclusions of our study.

Another relevant finding of the present study is that the factors associated with the risk of dying were almost the same at 1 and 3 years. As expected, mortality was higher for patients over 65 years of age, underweight according to BMI, not self-sufficient, and with a catheter as first vascular access. Moreover, mortality was lower in patients born in countries other than Italy probably due to the fact that these patients where younger than Italians (mean age 52 vs. 69 years).

In addition, for both 1-year and 3-year mortality, the comorbidity factors of greatest impact were, dementia or psychiatric disorders, heart disease, peripheral vascular disease, COPD, cancer, and liver disease. The observation that patient characteristics at the time of dialysis entry associated with mortality were almost the same for both 1-year mortality and 3-year mortality may suggest a longer-term effect of patient conditions at baseline.

Finally, variables associated with 1-year and 3-year mortality did not differ significantly among the three periods of incidence. Interestingly, in the study by Boenink et al. [1], ESRD patients starting HD showed a decrease in excess mortality for all causes of death with advancing calendar time, especially for atheromatous cardiovascular disease and infections. The RRDTL makes it possible to collect a lot of information about the incident dialysis patients that allows a precise description of individual socio-demographic characteristics and clinical conditions. These characteristics were considered in the models to obtain an adjusted estimate of the 1-year and 3-year mortality. However, residual confounding due to factors not retrievable in the RRDTL survey might play a role. One strength of this study is the inclusion of a large population with a long-term follow-up. To the best of our knowledge, this is one of the few studies that have recently evaluated early survival in a European population of dialysis patients. A further strength is the availability of a regional dialysis registry (RRDTL), which provides detailed data of all dialysis patients of Lazio region, that can be integrated by data from other regional healthcare databases, making it a powerful epidemiological tool to monitor survival rates.

## Conclusions

In summary, the present study showed that 1- and 3-year mortality in patients with end-stage renal disease starting HD in the Lazio region, was stable over 9 years. Investigation on longer term mortality (e.g., 5-year) may add information helpful to interpret the phenomenon. In the meantime, evidence from previous studies in the same context could be considered for potential improvements, such as an increased use of FAV which has been associated with better outcomes [17].

## Electronic supplementary material

Below is the link to the electronic supplementary material.

Supplementary Material 1

## Data Availability

Data related to the findings reported in our manuscript are available to all interested researchers upon request because of stringent legal restrictions regarding privacy policy on personal information in Italy (national legislative decree on privacy policy n. 196/30 June 2003). For these reasons, our dataset cannot be made available on public data deposition. All interested researchers can contact the following persons to request the data: Nera Agabiti, Department of Epidemiology, Lazio Regional Health Service, Rome, Italy, e-mail: n.agabiti@deplazio.it; Damiano Lanzi, Department of Epidemiology, Lazio Regional Health Service, Rome, Italy, e-mail: d.lanzi@deplazio.it.

## List of Abbreviations

ANOVA: Analysis of Variance
BMI: Body Mass Index
CI: Confidence Intervals
CMR: Crude Mortality Rate
COPD: Chronic Obstructive Pulmonary Disease
CVC: Catheter
ERA-EDTA: the European Renal Association Dialysis and Transplantation
ESRD: End Stage of Renal Disease
FAV: Fistula
HD: Hemodialysis
HR: Hazard Ratios
IQR: Interquartile Range
RRDTL: Lazio Regional Dialysis and Transplant Registry
UK: United Kindom
Vs: Versus
